# Perforation, Migration, and Omentum Embedding of a Levonorgestrel Intrauterine Device: A Case Report

**DOI:** 10.7759/cureus.67198

**Published:** 2024-08-19

**Authors:** Karen D Gómez-Arciniega, Erik D Palomares-Castillo, Héctor A Benítez-Jauregui, Jesús R Gastelum-Sarabia, Marcos D Ayala-López

**Affiliations:** 1 General Surgery, Centro Médico Nacional de Occidente, Instituto Mexicano del Seguro Social, Guadalajara, MEX; 2 General Surgery, Hospital General Regional No. 46, Instituto Mexicano del Seguro Social, Guadalajara, MEX

**Keywords:** intraperitoneal, migration, omentum, uterine perforation, levonorgestrel intrauterine device

## Abstract

The intrauterine device (IUD) is currently one of the most widely used methods due to its great effectiveness. Uterine perforation and migration of the device is one of its most serious complications, although rare. In most patients, it usually occurs at the time of placement and goes unnoticed; however, it can also occur late. The diagnosis is established by imaging studies, preferring abdominal ultrasound, and its treatment should be removal in all cases.

We present the case of a 27-year-old woman, with a history of levonorgestrel IUD placement two years earlier, who presented with chronic pelvic pain. During a gynecological consultation, the IUD threads were not evident. An abdominal CT scan showed that the IUD was in the abdominal cavity, so open abdominal surgery was performed where the IUD was found embedded in the omentum and the segment of the omentum containing the IUD was resected. The patient evolved satisfactorily and was discharged 24 hours after surgery.

## Introduction

The intrauterine device (IUD) is a method that is easy to apply, safe, effective, and commonly used for non-permanent contraception [1]. However, it is worth mentioning that the IUD has some rare complications that include infections, menstrual irregularities, contraceptive failure, and uterine perforation, the latter being the most serious complication with severe morbidity and may even be a cause of death [1].

Perforation usually occurs at the time of insertion and rarely occurs spontaneously during the puerperium [1,2]. Once perforation has occurred, the IUD can migrate and localize in various neighboring organs. Ectopic localization has been documented in the pouch of Douglas, the omentum, the mesentery, the colon, and even the bladder [3].

The World Health Organization (WHO) recommends the immediate removal of a displaced IUD, even in patients without symptoms and regardless of the type of IUD and its location [2,3]. Prior to surgery, the IUD should be accurately located by imaging [4].

## Case presentation

The patient is a 27-year-old woman, with no significant family history, chronic diseases, or surgical history, three pregnancies, three deliveries born at term and currently alive, and no abortions or cesarean sections (TPAL: 3.0.0.3). She had a levonorgestrel IUD inserted two years previously.

The patient had been suffering from intermittent chronic pelvic pain for the last 10 months, denied the presence of abnormal uterine bleeding, and had no other symptoms. Despite not having contraceptive failure, due to the presence of symptoms, she wanted to remove the levonorgestrel IUD and change her contraceptive method, so she went to consult with her gynecologist. However, during the gynecological examination, the IUD threads could not be visualized, so her gynecologist ordered an abdominal CT scan because of the suspicion of IUD migration.

**Figure 1:**
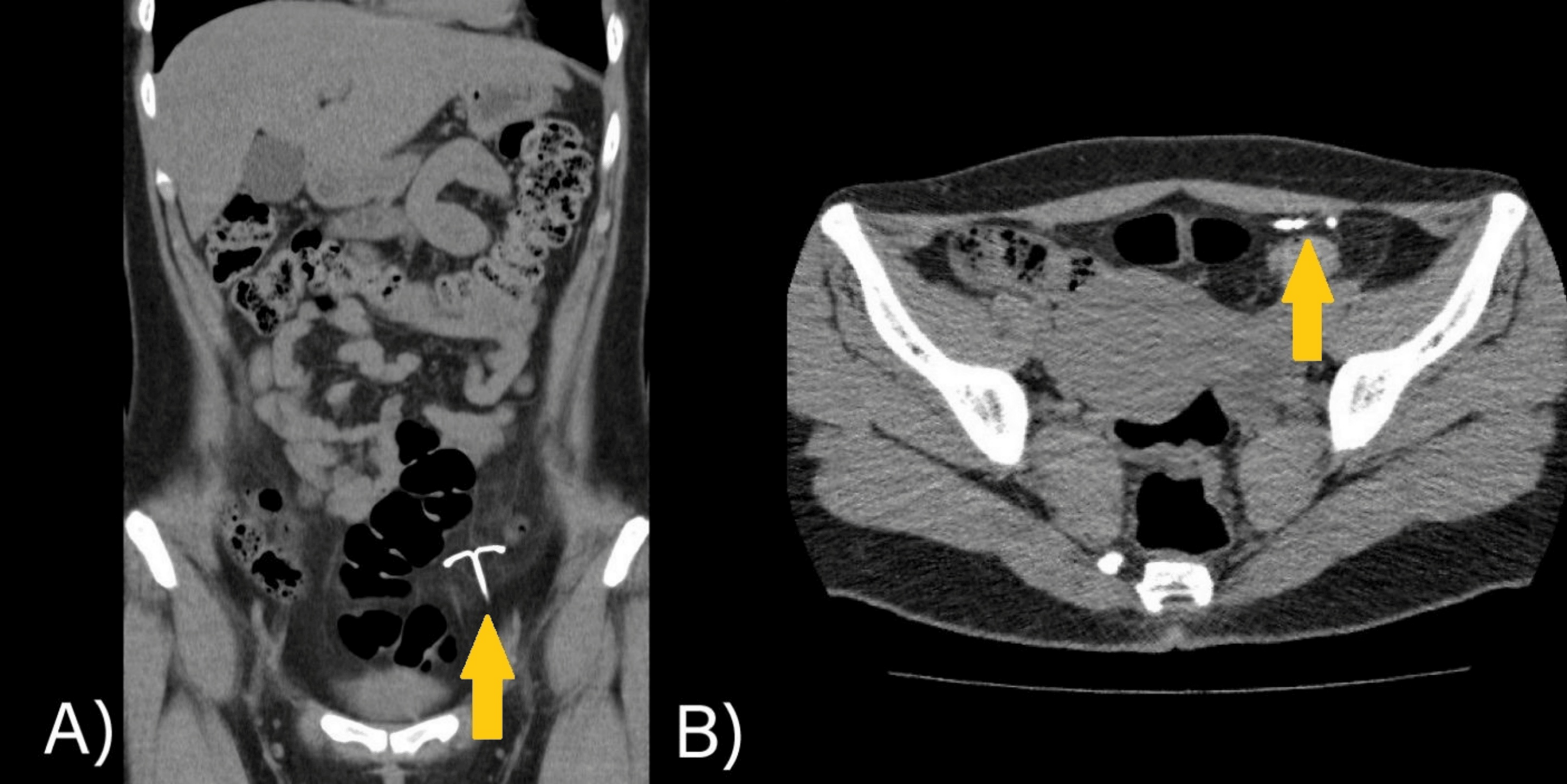
Abdominal CT scan: (A) coronal section and (B) axial section. The yellow arrows show the extrauterine location of the IUD, which is intra-abdominal in the left pelvic hollow. IUD: intrauterine device

The simple abdominal CT scan showed a T-type IUD translocation in the pelvic hollow, to the left of the midline, with no evidence of fluid or focal collections (Figure 1). For this reason, she was sent to the general surgery office, and it was decided to remove it in a programmed way by open surgery.

**Figure 2:**
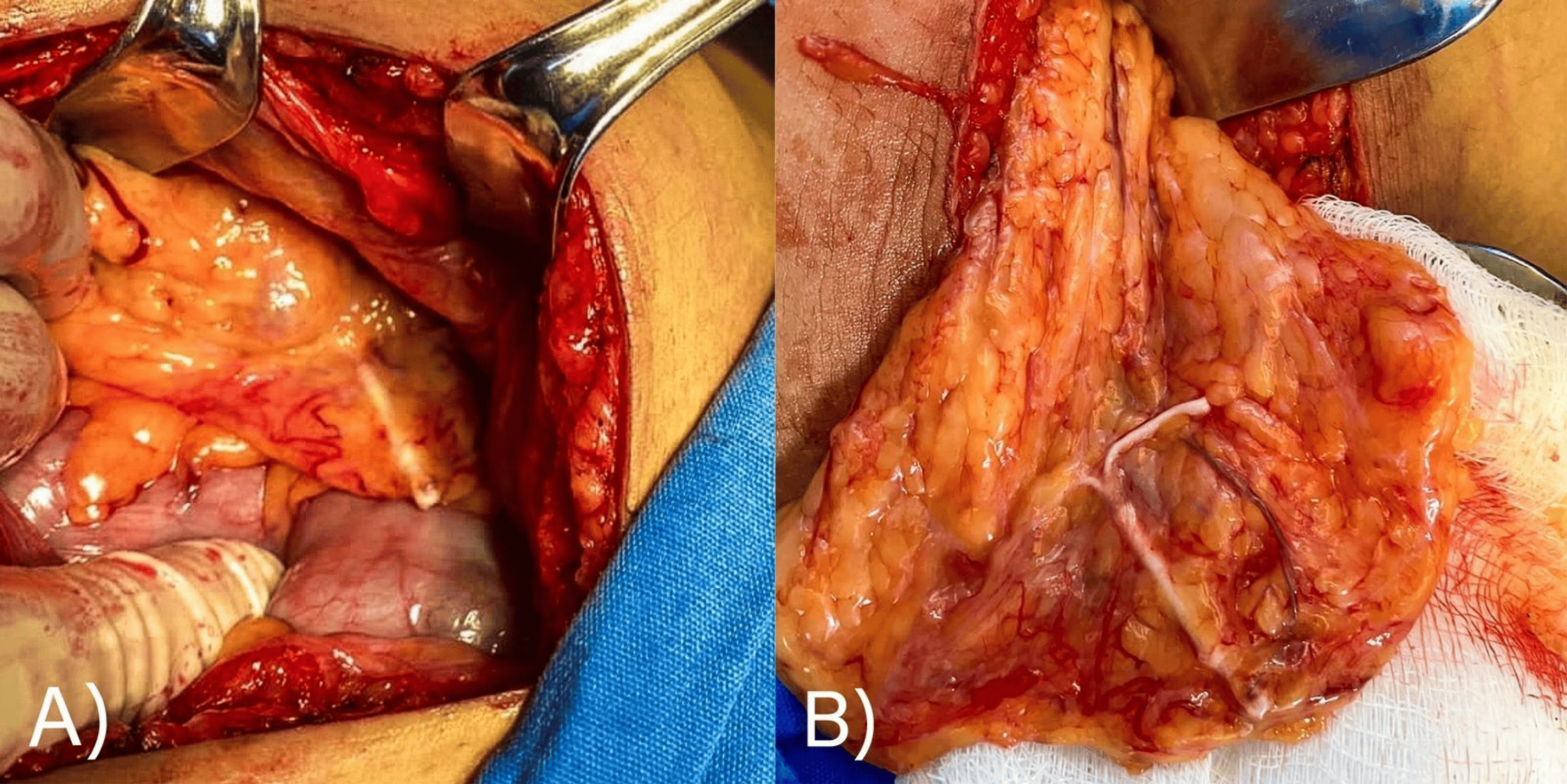
Intraoperative photograph: localization of intra-abdominal IUD. (A) Presence of the IUD embedded in a segment of the omentum. (B) The segment of the omentum containing the IUD is hypervascularized. IUD: intrauterine device

**Figure 3:**
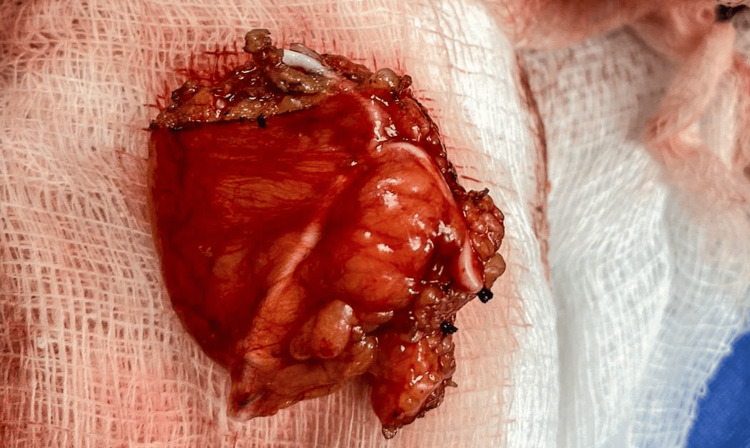
Surgical piece containing the foreign body. A 5×5 cm omentum containing the embedded IUD was removed. IUD: intrauterine device

An exploratory laparotomy-type surgical intervention was performed, through an infraumbilical incision. During surgery, the IUD was identified to be embedded in the omentum, which was already peritonized and with increased vascularity (Figure 2). No lesions were identified in the uterus, ovarian, colon, or rectum. It was decided to perform a partial omentectomy, so a 5×5 cm segment of the omentum containing the embedded IUD was resected (Figure 3). The piece was sent for histopathological study, which revealed no evidence of malignancy.

The patient was kept under hospital surveillance for 24 hours. She evolved satisfactorily, and there was no development of surgical site infection, seroma, or any other complication. Hence, the patient was discharged the following day, once she walked and tolerated a regular diet and intestinal transit was reestablished.

The patient was followed up by the general surgery outpatient clinic for one year, with no evidence of any surgical complications.

## Discussion

IUDs are a long-acting method, with high efficacy and safety and low cost, which has made them the most widely used reversible contraceptive option today [2,4]. However, complications can occur when using an IUD, of which some of the most common are as follows: being located in an unsuitable position, development of pelvic inflammatory disease, contraceptive failure, and uterine perforation [2]. Frameless copper-releasing IUDs are thought to be better tolerated and to present less risk of perforation [5].

Uterine perforation has an incidence rate of perforation of 1/1000 [2]. There are two instances when perforation can occur: firstly, at the time of insertion which is usually the most common form and is associated with the presence of severe abdominal pain, which should be an alarm for the health professional, and secondly, in a late form, due to progressive pressure and the development of uterine wall necrosis [2,3].

Once perforation has occurred, the clinical picture is not very clear, since most patients will be asymptomatic, while the rest may present with contraceptive failure, absence of threads during the gynecologic inspection, active bleeding, perforation or fistula of the bowel and bladder, or development of severe sepsis [2,4].

After perforation, the IUD can migrate and localize in various organs. Up to 80% of the devices will be found in the peritoneal cavity, while the rest will be located in the surrounding pelvic organs, such as the bladder, rectosigmoid bladder, appendix, omentum, small bowel, adnexa, and iliac vein [3]. Patients with IUD encrustation in the bowel and urinary tract may develop peritonitis [5].

Some risk factors for presenting this complication have been described in the literature, among them are a postpartum uterus, especially within six months, breastfeeding, retroverted uterus, uterus with small or irregular endometrial cavities, and little experience of the attending physician [5,6].

The first sign that should initiate the suspicion of perforation is the absence of threads [5]. Clinical diagnosis is not always possible. Imaging studies provide us with the advantage of being able to accurately identify the location of the migrated IUD. The first imaging option is abdominopelvic ultrasound [3]. However, there are other studies that can support the diagnosis, such as abdominal radiography, CT, and MRI [7].

The WHO recommends the surgical removal of the migrated IUD as soon as possible, even in asymptomatic patients. This is due to the risk of developing severe adhesions, chronic pain, and even infertility. Minimally invasive methods such as hysteroscopy, cystoscopy, colonoscopy, or laparoscopy are recommended, depending on where the IUD is located. However, if the IUD is embedded in any organ, exploratory laparotomy is recommended as the first option [1,3].

In the case of our patient, she had a history of levonorgestrel IUD placement two years ago and had been suffering from intermittent pelvic pain for 10 months. In addition to the gynecological examination, the IUD threads could not be visualized. According to the literature, the latter is usually the first sign of device migration. An abdominal CT scan corroborated the presence of the levonorgestrel IUD migration into the abdominal cavity.

As recommended by the WHO, we decided to surgically remove the migrated device in order to avoid complications. Open surgery was performed by exploratory laparotomy, where the IUD was found embedded in the omentum, the segment of the omentum containing the IUD was resected, and the patient had a satisfactory postoperative evolution.

## Conclusions

The IUD is a highly effective contraceptive method; however, one of its most rare and feared complications is uterine perforation and migration of the device to the different surrounding organs. In the case of clinical suspicion when the IUD threads are not identified, it is necessary to perform imaging studies to confirm the migration of the device, and if so, removal should be performed in all cases to avoid complications such as adhesions, pain, perforation of other organs, or infertility. The removal can be performed by laparoscopy or other minimally invasive means; however, if there is suspicion of an IUD embedded in any organ, it is recommended to opt for an exploratory laparotomy.
